# Extrapontine myelinolysis associated with pituitrin: case report and literature review

**DOI:** 10.1186/s12883-014-0189-9

**Published:** 2014-10-09

**Authors:** Liying Zhuang, Ziqi Xu, Yaguo Li, Benyan Luo

**Affiliations:** Department of Neurology, The First Affiliated Hospital, College of Medicine, Zhejiang University, Hangzhou, 310003 Zhejiang China; Department of Neurology, Zhejiang Hospital, Hangzhou, 310013 Zhejiang China

**Keywords:** Pituitrin, Hyponatremia, Extrapontine myelinolysis

## Abstract

**Background:**

Hyponatremia is the most common electrolyte abnormality encountered in hospitalized patients, resulting from a varied spectrum of conditions. Both the primary disturbance and its correction can result in life-threatening neurological consequences. Extrapontine myelinolysis is one such complication that is associated with the rapid correction of hyponatremia. Here we describe a patient who developed extrapontine myelinolysis unexpectedly after the correction of hyponatremia, which involved the drug pituitrin.

**Case presentation:**

A 24-year-old Chinese woman was transferred to our neurology department with the symptoms of dysarthria and quadriparesis developing one day after the correction of hyponatremia (from 118 mmol/L to 140 mmol/L), which followed with a continuous intravenous drip of pituitrin used to control hemoptysis in the emergency room. During the course, she developed involuntary movement. Magnetic resonance imaging changes were consistent with extrapontine myelinolysis.

**Conclusion:**

This present case describes the mechanism of profound hyponatremia involving pituitrin, and the subsequent development of extrapontine myelinolysis. Physicians may approach effective clinical management of patients through awareness of the adverse effect of pituitrin on serum sodium levels, and avoid rapid correction of hyponatremia in clinical practice.

## Background

Hemoptysis, a common symptom in clinical practice, can sometimes become a life-threatening situation and require urgent management. Pituitrin is the best available drug for the control of severe pulmonary haemorrhage or repeated hemoptysis due to its ability of strong vasoconstriction [1]. Untoward effects of pituitrin, such as hyponatremia, are often ignored by physicians, as most patients with hyponatremia are asymptomatic. Hyponatremia is generally defined as plasma sodium level of less than 135 mmol/L [2]. Although hyponatremia is a common and often underestimated problem, rapid correction of chronic hyponatremia can have devastating neurological consequences, i.e., central pontine myelinolysis (CPM) and extrapontine myelinolysis (EPM). CPM and EPM are two variants of osmotic demyelination syndrome, which is related to rapid osmotic changes, particularly an aggressive correction of hyponatremia [3]. EPM may involve the cerebellum, lateral geniculate body, basal ganglia and cerebral white matter with varied spectrum of symptoms. Parkinsonism is common, while involuntary movements such as dystonia and myoclonus are less frequently observed [3,4]. We report a case of iatrogenic EPM presenting with dysarthria, quadriparesis and involuntary movements following correction of hyponatremia associated with pituitrin.

## Case presentation

A 24-year-old Chinese woman was transferred to our neurology department presenting with dysarthria and quadriparesis. The patient had a medical history of tetralogy of Fallot since infancy with resulting cyanosis. Corrective surgery was undertaken 4 years ago, and she had been stable and functionally well since then. She had no other comorbid conditions such as alcoholism, malnutrition, hepatic cirrhosis or renal insufficiency. The episode began when the patient contracted repeated minor hemoptysis, she received conventional therapy with aminomethylbenzoic acid and etamsylate without efficiency. On the fifth day, she began to get a fever and was transferred to the emergency room in our hospital.

**Figure 1:**
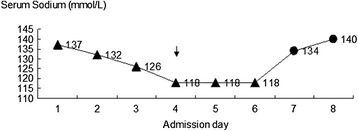
**Changes of the patient’s serum sodium concentration.** Triangles showing the serum sodium gradually decreased with the use of pituitrin, the black arrow pointing out the day beginning use of hypertonic saline.

**Figure 2:**
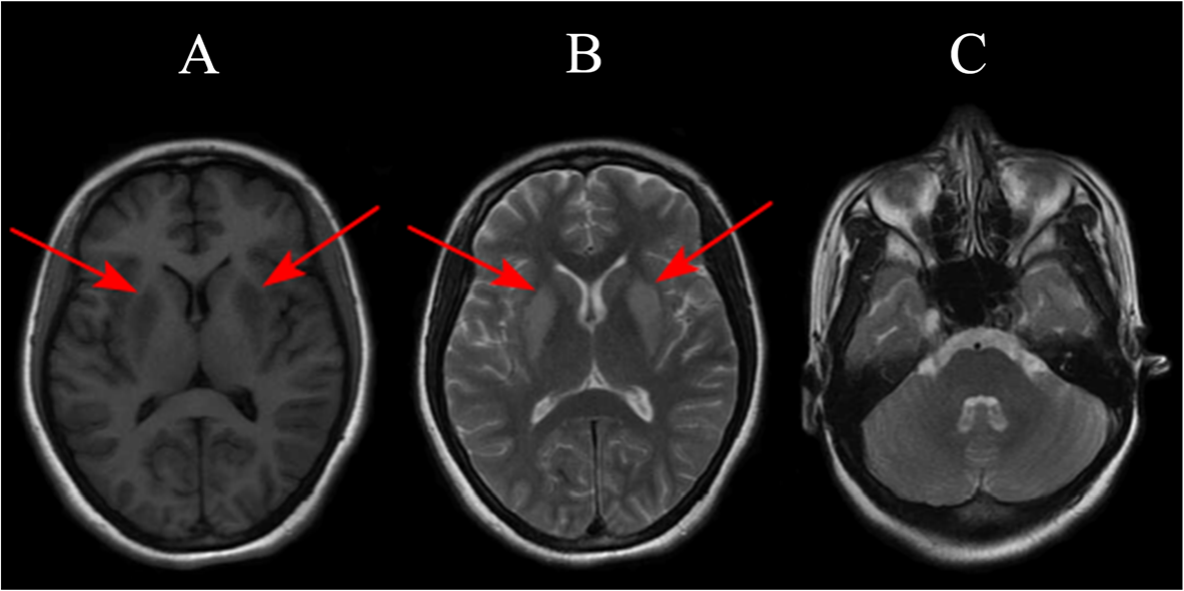
**Brain magnetic resonance imaging. A**, axial T1-weighted MRI showing low signal intensity of bilateral symmetric basal ganglia; **B**, axial T2-weighted MRI showing high signal intensity of bilateral symmetric basal ganglia; **C**, axial T2-weighted MRI showing no lesion in the pons (the red arrows pointing out the lesions).

On admission, her symptoms worsened with a massive hemoptysis, and pituitrin was prescribed to control the bleeding. Further assessment of the origin of bleeding by computed tomography (CT) angiography of the chest showed that the right bronchial artery was slightly dilated, and a multidisciplinary consultation suggested expectant treatment with pituitrin instead of bronchial artery embolization. Pituitrin (18 U in 30 ml of saline) was administered by intravenous drip continuously at a rate of 2 ~ 3 ml/h via a pump for six days. On the fourth admission day, the patient became nauseous and exhibited generalized weakness. Blood tests revealed a profound hyponatremia with a sodium level of 118 mmol/L, a serum osmolality of 253 mOsm/kg H2O, and hepatic and renal functions were normal. Other biochemical tests showed normal thyroid function, and serum cortisol and adrenocorticotropic hormone were within the normal range. The patient was resuscitated with i.v. hypertonic saline, and the sodium level was 140 mmol/L four days later (Figure 1). Unfortunately, the next day she deteriorated once more, initially developing dysarthria and quadriparesis. An immediate CT of the head presented normal, and she was transferred to our neurology department. During the course, she developed involuntary movements, mainly paroxysmal oromandibular dystonia and myoclonus in the left upper limb. Magnetic resonance imaging (MRI) showed bilateral symmetric basal ganglia lesions consistent with EPM (Figure 2). She was treated mainly with corticosteroid, diazepam and hyperbaric oxygen therapy and was discharged with few residual symptoms (speaking with relative fluency and walking on her own without involuntary movements).

## Conclusions

This patient developed EPM unexpectedly after the intravenous use of pituitrin to control hemoptysis. Pituitrin, extracted from the posterior pituitary, consists of oxytocin and vasopressin. The latter can activate type-1A receptors located in vascular smooth muscle cells resulting in vasoconstriction [5]. Thus, pituitrin has been used in gastrointestinal or pulmonary haemorrhage, and the result is almost as if forceps had been applied directly to the bleeding vessel. In addition, water reabsorption is mediated by vasopressin activation of type-2 receptors in the basolateral membrane of cells in the renal collecting ducts, which causes a decrease in plasma osmolality [5]. There was no medical history of hyponatremia, absence of adrenal, thyroid, pituitary or renal insufficiency, and no recent use of diuretic agents in this patient; pituitrin was implicated as the very probable contributory factor to the development of severe hyponatremia.

Hyponatremia can be classified into acute (<48 h) and chronic (≥48 h) hyponatremia. When hyponatremia develops, the brain reduces the number of osmotically active particles within its cells (mostly organic solutes) in an attempt to adapt to the osmotic change, which takes 48 h [6]. CPM and EPM are acquired metabolic disorders of acute central demyelination strongly associated with rapid correction of hyponatremia [7]. CPM typically involves the central pontine and EPM involves different brain regions, such as the cerebellum, thalamus, basal ganglia or subcortical white matter. Clinical heterogeneity due to CPM/EPM affecting the basal ganglia has been reviewed by de Souza A [8], such as dystonia, tremor, myoclonus, gait disorders, dysarthria, cognitive impairment, depression and others. Focal oromandibular dystonia and asymmetric myoclonus that developed in a delayed manner in the patient are rare presentations. The possibility that delayed movement disorders may arise in EPM due to ineffective or faulty reorganization in the basal ganglia has been considered in an earlier report [9]. Sequential observation of symptoms and brain images in the case of CPM and EPM revealed that delayed movement disorders as a result of changes in the signal of the basal ganglia, were explained by the destruction of regional myelin [10]. Special populations seem vulnerable to the development of osmotic myelinolysis, including not only alcoholics and malnourished patients but also those with liver disease, sepsis, adrenal insufficiency and severe burns [11]. Previous cases have shown that the chronicity of hyponatremia before correction is a critical risk factor for the development of osmotic myelinolysis [12,13]. In this patient, chronic hyponatremia was documented to exist for at least 48 h, and after a rapid increase of serum sodium from 118 mmol/L to 134 mmol/L in 24 h, there was a delay of one day before progressive neurological decline. Recommendations suggest that ideally hyponatremia should be corrected by limiting the sodium increase to not more than 8–10 mmol/L every 24 h [2]. We identified three cases, each reporting a single case in which deamino arginine vasopressin, a type of vasopressin analogue, was implicated as a possible contributory factor to the development of severe electrolyte imbalances that triggered osmotic demyelination [14-16]. Although the true aetiology of CPM/EPM remains unclear, abrupt osmotic shifts have been considered to play a critical role in their pathogenesis. Deamino arginine vasopressin and pituitrin may predispose patients to osmotic demyelination by causing hyponatremia and extreme serum sodium fluctuations rather than by exerting any direct myelinolytic effect. Further studies are needed to clarify whether the use of pituitrin or its analogue is associated with a risk of osmotic demyelination independent of the electrolyte disturbance.

This case and literature review highlight that: 1) Hyponatremia is the most frequent electrolyte disturbance observed in hospitalized patients [17]. Drugs such as pituitrin can cause profound hyponatremia, which should be considered in the differential diagnosis when approaching a patient with hyponatremia. 2) Osmotic myelinolysis is most frequently associated with rapid correction of hyponatremia [3]. Prevention of CPM and EPM by exercising caution in the correction of hyponatremia, especially chronic hyponatremia, is more important than early diagnosis.

### Consent

Written informed consent was obtained from the patient for publication of this case report and any accompanying images. A copy of the written consent is available for review by the Editor of this journal.

## Abbreviations

CPM: Central pontine myelinolysis
EPM: Extrapontine myelinolysis
CT: Computed tomography
MRI: Magnetic resonance imaging
